# Radio-laboratory findings in COVID-19 anosmia patients: a single-center experience

**DOI:** 10.1186/s43163-021-00133-6

**Published:** 2021-07-29

**Authors:** Tareq Muhammad Algarf, Mahmoud Atef Youssef, Mostafa Elshazly, Mohamed Said Ismail, Mohamed Kamal Hasswa, Mohamed Shaaban Mousa

**Affiliations:** 1 Department of ENT, Faculty of Medicine, Cairo University, Giza, Egypt; 2 Department of Pulmonary Medicine, Faculty of Medicine, Cairo University, Giza, Egypt

**Keywords:** Covid 19, Anosmia, Olfactory dysfunction, Anosmia in covid, Anosmia in corona virus

## Abstract

**Background:**

There is solid evidence that olfactory dysfunction (OD) can present in COVID-19 patients. Anosmia can be the only presentation or can be accompanied by other symptoms of COVID-19. Multiple cross-sectional studies have demonstrated that the incidence rate of olfactory dysfunction is high in COVID-19 patients with good prognosis. The aim of our study is to investigate the presence of OD with the radiologic and laboratory findings among COVID-19 positive patients.

**Results:**

There was statistical significance in clinical severity between anosmia and non-anosmia group (*P* value 0.000) denoting that anosmia sign mostly occur in mild COVID. Also, there was significance in D dimer between two groups (*P* value 0.044) denoting that D dimer could be a sign of clinical severity and it is usually not elevated in anosmia. All anosmia group had normal CT chest denoting that it is a mild form of COVID-19.

**Conclusions:**

Olfactory dysfunction (OD) is an imminent sign of COVID-19 patient, which can be presented as a sole symptom or with other symptoms. As anosmia could be the sole clinical presentation of COVID-19 patients without any other significant signs and so otolaryngologists should be aware of this presentation in COVID-19 diagnosis.

## Background

Coronavirus disease 2019 (COVID-19) is on-going pandemic started in Wuhan, China, in December 2019. It is caused by novel enveloped single-stranded ribonucleic acid (RNA) beta-coronavirus, which is known as the severe acute respiratory syndrome coronavirus 2 (SARS-CoV-2) [1].

To date, over 108.2 million COVID-19 cases and 2.3 million deaths have been reported to WHO till February 2021 [2].

COVID symptoms range from mild symptoms to severe illness. Symptoms may appear 2-14 days after exposure to the virus. Typical symptoms include fever, cough, and shortness of breath [3].

A team at King’s College, London, added loss of sense of smell to the COVID-19 symptom tracker. And it is a stronger predictor of coronavirus infection than fever [4].

In 17th April 2020, Center of Disease Control (CDC) set isolated anosmia as COVID-19 symptom followed by WHO in May 2020 [5].

In COVID-19 positive patients, 59% reported losing their sense of smell or taste, compared with only 18% testing negative. So, anosmia is an early warning sign of infection, and patients should be self-isolated to reduce the spread of the disease [4].

Olfactory dysfunction often accompanied by dysgeusia in COVID-19 patients, suggesting a probable association between the two [6].

A lot of studies correlate anosmia with the severity of COVID-19 illness. Mayo clinic systematic review suggested that OD may be associated with a milder course of COVID-19 infection [7].

Novelty of our study is to investigate the presence of OD with the radiologic and laboratory findings among COVID-19 positive patients.

## Methods

Our study included total of 146 patients that were admitted to our institute. All patients were either mild or moderate COVID-19 that were admitted in inpatient wards.

Approval of the local ethical committee of the hospital was obtained to conduct the current study. Besides that, written informed consents were obtained from all participants clarifying the purpose of the study conforming Helsinki Declarations (1964).

In the absence of full objective smell assessment and nasopharyngoscope (contraindicated in the current situation), it is acceptable to depend on subjective self-assessment of olfactory dysfunction [8].

All included patients have been subjected to full medical history taking and were proven to be COVID-19 positive by PCR (nasopharyngeal swab). Prior to admission, all patients performed computed tomography (CT) of the chest and laboratory investigations in the form of CBC with differential count, ALT, AST, ferritin, CRP, D dimer, urea, and creatinine and detection of oxygen saturation by pulse oximetry. According to this initial assessment, severity of the condition could be determined.

Severity was classified into mild, moderate, or severe.

Mild type means mild symptoms in the absence of any radiological abnormalities in CT chest, moderate type includes fever and respiratory tract symptoms, and pneumonia is observed on imaging while severe type include any of the following criteria are met: respiratory distress as evidenced by respiratory rate ≥ 30 breaths/min, oxygen saturation ≤ 93% at rest, and arterial blood oxygen partial pressure/oxygen concentration ≤ 300 mmHg with the lesions significantly progressing > 50% within 24–48 h on pulmonary imaging [9].

To be enrolled in the study, patients had to meet the following inclusion criteria: adults over 18 years of age, nasopharyngeal swab positive for COVID patients and acceptance for participation in the study.

On the other hand, the study exclusion criteria were uncooperative patients, patients on ventilators, psychiatric or neurological disorders, previous surgery, and radiation or trauma in nasal cavity.

A rapid office-based screening of smell disorder can be done, using a subjective olfaction score. The patient is asked to estimate his sense of smell on a 10-point scale bar with 0: anosmia, 10: normal smell and scores from 1 to 9 mean progressive increase in the degree of smell toward normal. As olfactory affection is a pertinent feature of COVID-19, ENT surgeons should pay attention to this particular disorder during patients’ examination. The use of subjective olfaction score for rapid screening of patients may be beneficial to early diagnose the disease and prevent its spread [8].

We investigated the presence of OD with the radiologic and laboratory findings among COVID-19 positive patients.

### Statistical analysis

Data was entered and statistically analyzed on the Statistical Package of Social Science Software program, version 25 (IBM SPSS Statistics for Windows, Version 25.0. Armonk, NY: IBM Corp.). Data was presented using range, mean, standard deviation, median and interquartile range for quantitative variables, and frequency and percentage for qualitative ones. Comparison between groups for qualitative variables was performed using Chi square or Fisher’s exact tests while for quantitative variables the comparison was conducted using Mann-Whitney test. P values less than or equal to 0.05 were considered statistically significant.

## Results

The current study included a total of 146 COVID-19 positive patients who were laboratory confirmed by RT-PCR (nasopharyngeal swab), and were admitted to the hospital. All participants were Egyptians with mean age 38.34 ± 11.72 including 100 males (68.5%) and 46 females (31.5%) denoting male gender susceptibility to COVID-19 (Table 1).

**Table 1:** Demographic, laboratory, clinical, and radiologic assessment of patients

	Description (*n* = 146)
**Age**	
Range	22-68
Mean ± SD	38.34 ± 11.72
Median (IQR)	38 (27-47)
**Sex**	
Male	100 (68.5)
Female	46 (31.5)
**Occupation**	
Medical	98 (67.1)
Non-medical	48 (32.9)
**Smoking**	
Smoker	34 (23.3)
Non-smoker	112 (76.7)
**Baseline ferritin**	
Range	7.3-1898
Mean ± SD	285.94 ± 340.6
Median (IQR)	175.7 (62-345)
**D dimer**	
Range	0.1-4.43
Mean ± SD	0.53 ± 0.68
Median (IQR)	0.33 (0.22-0.48)
**CRP**	
Range	0.2-89
Mean ± SD	12.08 ± 16.66
Median (IQR)	5 (2.2-15.9)
**Lymphopenia**	
Yes	50 (34.2)
No	96 (65.8)
**Nasal**	
No	116 (79.45)
Anosmia	30 (20.54)
**Severity**	
Mild	84 (57.5)
Moderate	62 (42.5)

Our patients were divided into two groups according to presence or absence of anosmia (Table 2).

**Table 2:** Presence or absence of anosmia

	Anosmia		
	Yes (***n*** = 30)	No (***n*** = 116)	***P*** value
**Age**			
Range	25-54	22-68	
Mean ± SD	36.13 ± 9.26	38.91 ± 12.24	
Median (IQR)	31 (28-46)	38 (27-49)	0.528
**Sex**			
Male	24 (80)	76 (65.5)	0.128
Female	6 (20)	40 (34.5)	
**Occupation**			
Medical	24 (80)	74 (63.8)	0.092
Non-medical	6 (20)	42 (36.2)	
**Smoking**			
Smoker	8 (26.7)	26 (22.4)	0.623
Non-smoker	22 (73.3)	90 (77.6)	
**Baseline ferritin**			
Range	22.4-814	7.3-1898	
Mean ± SD	239.38 ± 220.35	297.98 ± 365.13	
Median (IQR)	174 (82.9-287)	178.3 (55-352.4)	0.969
**D dimer**			
Range	0.13-0.84	0.1-4.43	
Mean ± SD	0.32 ± 0.17	0.59 ± 0.75	
Median (IQR)	0.27 (0.2-0.33)	0.35 (0.23-0.5)	0.044
**CRP**			
Range	0.2-36.7	0.3-89	
Mean ± SD	7.65 ± 8.71	13.23 ± 18.01	
Median (IQR)	6.2 (2.2-9.6)	4.5 (2.2-20)	0.410
**Lymphopenia**			
Yes	12 (40)	38 (32.8)	0.456
No	18 (60)	78 (67.2)	
**Severity**			
Mild	26 (86.7)	58 (50)	0.000
Moderate	4 (13.3)	58 (50)	

In Table 1, patients were 146 and they were divided according to their symptoms into the following: 98 patients had no nasal symptoms and 30 patients had anosmia.

**Fig. 1:**
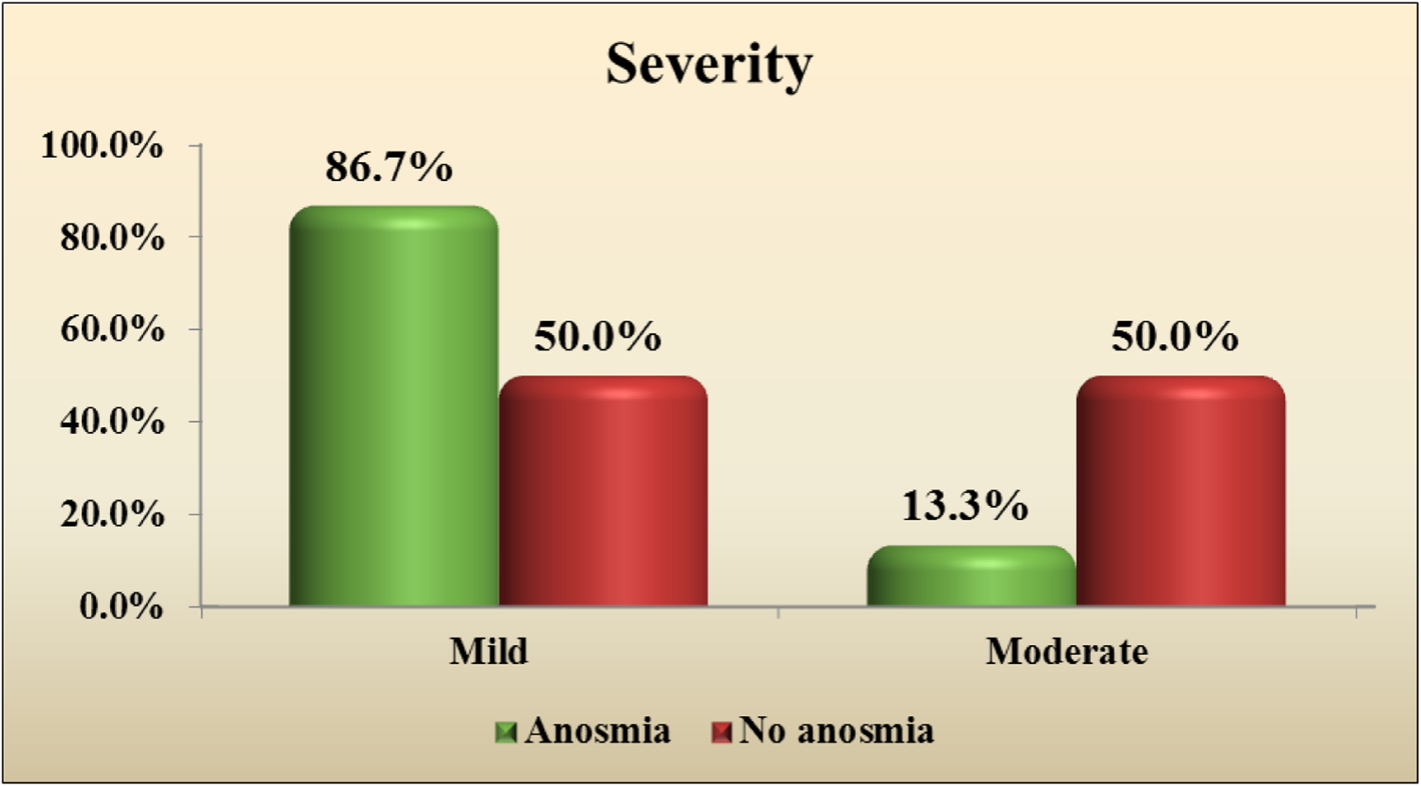
Clinical severity in anosmia and non-anosmia group

We found statistical significance in clinical severity between anosmia and non-anosmia group (*P* value 0.000) denoting that anosmia sign mostly occur in mild COVID (Fig. 1) (Table 2). Also, there was significance in D dimer between two groups (P value 0.044) denoting that D dimer could be a sign of clinical severity and it is usually not elevated in anosmia.

## Discussion

Olfactory neurons are the entry gate for neuro-invasion by coronavirus, which may be transferred to the central nervous system via a synapse-connected route. Still it is unclear whether olfactory sensory neurons are directly involved in the pathogenesis of smelling loss in COVID-19. SARS-CoV-2 virus has a spiny protein S1, which makes the virion adhere to the cell membrane by interacting with the host angiotensin-converting enzyme 2 (ACE2) receptor [6].

ACE2 is a functional receptor for SARS-CoV-2, and its expression and distribution in the nervous system suggest that SARSCoV-2 can cause neurological manifestations through direct or indirect pathways [6].

There is strong evidence that the nasal cavity is a crucial area susceptible to SARS-CoV-2 infection [10].

Viral loads in the patient’s nasal cavity were higher than that present in the pharynx, both symptomatic individuals and asymptomatic ones, hinting the nasal cavity as nasal mucosa may be the initial site of SARS-CoV-2 infection, in which the mode of transmission of SARS-CoV-2 is through infectious droplets [10].

However, COVID-19 case series reports showed a high rate of recovery of olfaction within 1-2 weeks after the onset of the dysfunction [11].

For these reasons, it is reasonable to put a hypothesis that the olfactory disorders (OD) are not related to, directly or indirectly, definitive viral damage to the neuronal cells. Conversely, the virus target may not be the neurons but other non-neuronal cells that express ACE2 receptors such as the olfactory epithelium sustentacular cells, microvillus cells, Bowman’s gland cells, horizontal basal cells, and olfactory bulb pericytes [12].

Brann et al. supposed that the anosmia reported by COVID-19 patients is due to the infection of the supporting cells and vascular pericytes of the olfactory epithelium and bulb which, consequently, alters the function of the olfactory neurons [12].

Several cross-sectional studies about the prevalence rate of OD in COVID-19 patients have been released in many countries through non-contact methods such as online questionnaires and telephone interviews [13–15].

This study is novel as it correlates anosmia to radio-laboratory findings in COVID-19 patients.

Along with our study, incidence of OD in COVID-19 patients was around 25% in these studies [12–16].

The studies also showed that individuals with smell disorders tend to have a taste disorder, suggesting a probable association between the two symptoms [7, 12–16].

Contrary to our results, most studies have found that the incidence of smell disorders in COVID-19 patients is higher in females than males [7, 11, 14]. In Mayo clinic systematic review, no gender difference was observed [7].

In Hornuss D et al., mean age of anosmic patients was 56 years, total leukocytic count was 6.3, lymphocyte was 0.8, CRP was 60 (mg/L), and shortness of breath was in 65% of our patients study.

Our study mean age was 36 years; total leukocytic count was 6.6 while lymphocytic count was 0.2. Moderate severity was found in 13% of patients.

Along with Mayo clinic systematic review [7], most of cases were mild in severity and CT chest was normal in majority of cases.

Majority of cases were of normal D dimer, leukocytic count, and lymphocytic count with no available studies to compare with.

### Limitations

The subjective olfaction score is not a meticulous scoring. Psychophysical tests for evaluation of smell such as the Sniffing’ Sticks test (Burghart; Wedel, Germany) and the University of Pennsylvania Smell Identification test (Sensonics Inc., Haddon Heights, NJ) are more reliable in the evaluation of olfaction, but they are not usually available during general health examination, besides being time consuming and costly.

The lack of consistent follow-up of our patients limits us from calculating the recovery time olfactory and gustatory functions, and, therefore, the percentage of permanent anosmia or ageusia.

## Conclusions

OD is a prominent finding in COVID-19 patient, which can occur independently or with other symptoms, but its pathogenesis is not well understood. Otolaryngologists should be aware of anosmia as a presentation of COVID-19 infection to avoid delaying the diagnosis of COVID-19. As anosmia could be the sole clinical presentation of COVID-19 patients without any other significant signs, it pushes otolaryngologists not to delay COVID-19 diagnosis. To avoid cross-infection, the otolaryngologist may consider a remote olfactory evaluation for COVID-19 patient with OD.

## Data Availability

The data of the study are available at the corresponding author upon request.

## Abbreviations

OD: Olfactory dysfunction
COVID-19: Coronavirus disease 2019
SARS-CoV-2: Severe acute respiratory syndrome coronavirus 2
CDC: Center of Disease Control
PCR: Polymerase chain reaction
ACE2: Angiotensin-converting enzyme 2
